# Habit Breaking Appliance for Multiple Corrections

**DOI:** 10.1155/2013/647649

**Published:** 2013-10-03

**Authors:** Reji Abraham, Geetha Kamath, Jasmeet Singh Sodhi, Sonia Sodhi, Chandki Rita, S. Sai Kalyan

**Affiliations:** ^1^Department of Orthodontics & Dentofacial Orthopedics, Sri Hasanamba Dental College and Hospital, Hassan, Karnataka, India; ^2^Department of Oral Medicine & Radiology, Sri Hasanamba Dental College and Hospital, Hassan, Karnataka, India; ^3^Department of Orthodontics & Dentofacial Orthopedics, CSMSS Dental College & Hospital, Aurangabad, India; ^4^Department of Oral Medicine, Diagnosis and Radiology, CSMSS Dental College & Hospital, Aurangabad, India; ^5^Department of Conservative Dentistry and Endodontics, Sri Aurobindo College of Dentistry, Indore, Madhya Pradesh, India; ^6^Department of Conservative Dentistry and Endodontics, Rural Dental College, Loni, Maharashtra, India

## Abstract

Tongue thrusting and thumb sucking are the most commonly seen oral habits which act as the major etiological factors in the development of dental malocclusion. This case report describes a fixed habit correcting appliance, *Hybrid Habit Correcting Appliance* (HHCA), designed to eliminate these habits. This hybrid appliance is effective in less compliant patients and if desired can be used along with the fixed orthodontic appliance. Its components can act as mechanical restrainers and muscle retraining devices. It is also effective in cases with mild posterior crossbites.

## 1. Introduction

Parafunctional habits are recognized as a major etiological factor for the development of dental malocclusion. Thumb sucking and tongue thrusting are the most commonly seen oral habits [1–3]. The line of treatment for these habits includes removal of the etiology, retraining exercises and use of mechanical restraining appliances [4, 5]. Tongue bead appliances are commonly used as retraining exercise devices [6–9]. In severe tongue thrusting cases and in cases with anterior open bite, a bead appliance alone may not be effective in restricting the habit. 

Tongue crib appliances are extremely effective in breaking the tongue thrust habit [4, 5, 10, 11]. They create a mechanical barrier and prevent the tongue from thrusting between the incisors. In most of the cases with severe thumb/digit sucking habit, an anterior open bite develops [12, 13]. This will result in the development of a secondary tongue thrust habit. Hence, in cases with severe prolonged thumb or digit sucking, an appliance which can eliminate both of these habits is appropriate [14].

Patient compliance is another problem associated with removable habit breaking appliances [15, 16]. Hence, habit breaking appliances which can be used along with fixed orthodontic appliances will be of great advantage.

This paper describes a habit breaking appliance *Hybrid Habit Correcting Appliance (HHCA)* which can be used to effectively restrain and correct tongue thrusting as well as thumb sucking habit. 

## 2. Appliance Design

**Figure 1:**
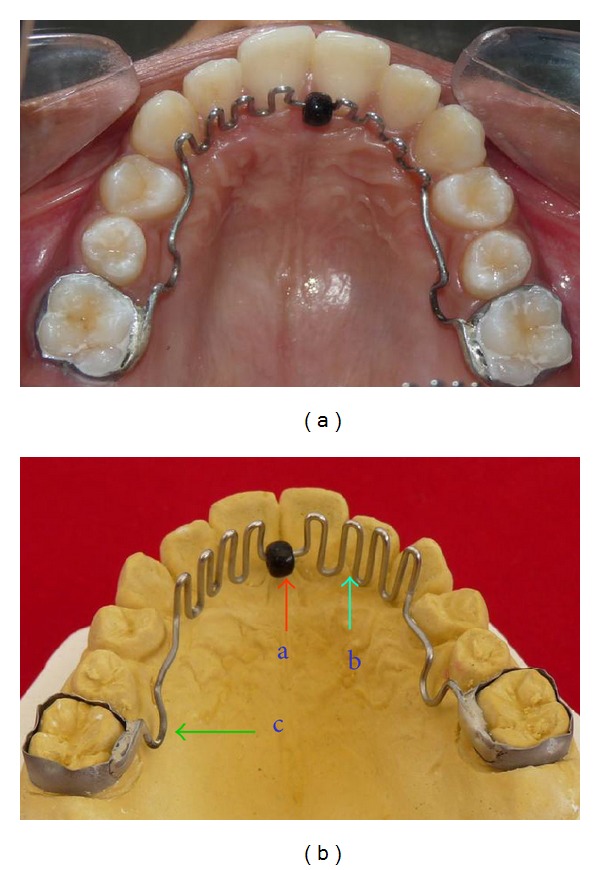
Hybrid Habit Correcting Appliance (HHCA).

*Hybrid Habit Correcting Appliance (HHCA) *(Figure 1) incorporates a tongue bead, a palatal crib and a U-loop which is attached to the molar bands on either sides.

The *tongue bead *(Figure 1(a)) consists of a spinnable acrylic bead of 3 mm diameter. The appliance is designed to position the acrylic bead over the posterior one-third of the incisive papilla. The bead acts as a tongue retrainer. The patient is asked to constantly pull the bead towards the posterior region of the mouth. The patient is also advised to make sure that his tongue wedges between the bead and the roof of the mouth as he swallows.

The palatal crib (Figure 1(b)) and the U-loop are made of 0.9 mm stainless steel wire. Three to four spurs are bent on either sides of the bead, starting from the canine region on one side, running anteriorly as a smooth curve (in conventional crib appliances, the cribs run obliquely from one canine to the other side canine) and lying 1 mm lingual to the cervical margin of the maxillary anterior teeth. In the region of the incisive papilla, the acrylic bead is incorporated in such a way that it lies over the posterior one-third of the incisive papilla. The tip of the crib should be almost in line with the incisor tip of the maxillary central incisor or 2 mm longer without interfering with the lower incisors when in occlusion. In cases with anterior open bite, the crib should be longer and can be up to 3/4th of the interincisal distance between the upper and lower central incisors. This is to avoid the tongue from thrusting over the tip of the crib. The palatal crib acts as a barrier against the thrusting tongue and works as a mechanical restrainer. 

The U-loop (Figure 1(c)) is incorporated in the second premolar region and it helps to reposition the appliance posteriorly during the retraction phase, when it is used along with fixed orthodontic appliances.

**Figure 2:**
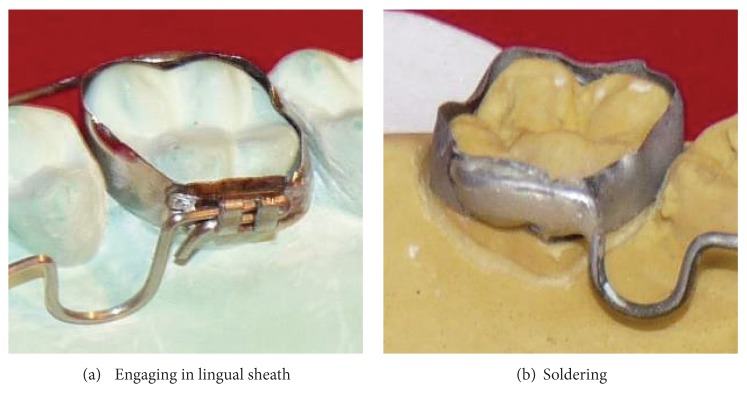
Appliance secured to molar band by (a) engaging in lingual sheath and (b) soldering.

The appliance can be engaged into a lingual sheath (Figure 2(a)) on the molar bands or can be soldered directly to the molar band (Figure 2(b)). If it is engaged in a lingual sheath, a tight ligature tie should be wound around the lingual sheath and the distal end of the appliance to avoid the appliance from slipping out of the sheath into the oral cavity.

## 3. Case Report

**Figure 3:**
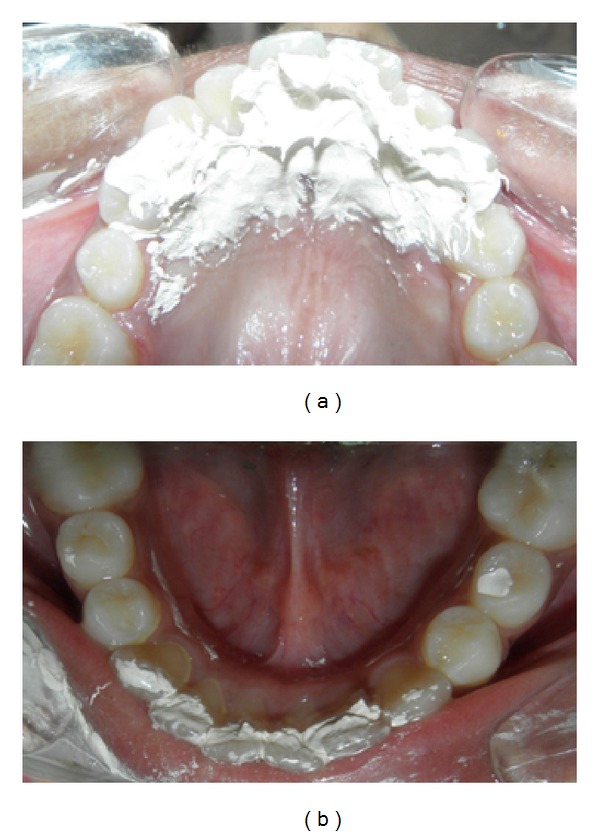
Pretreatment palatographic analysis showing impression material smeared over the maxillary and mandibular anteriors indicating abnormal tongue posture during swallowing.

**Figure 4:**
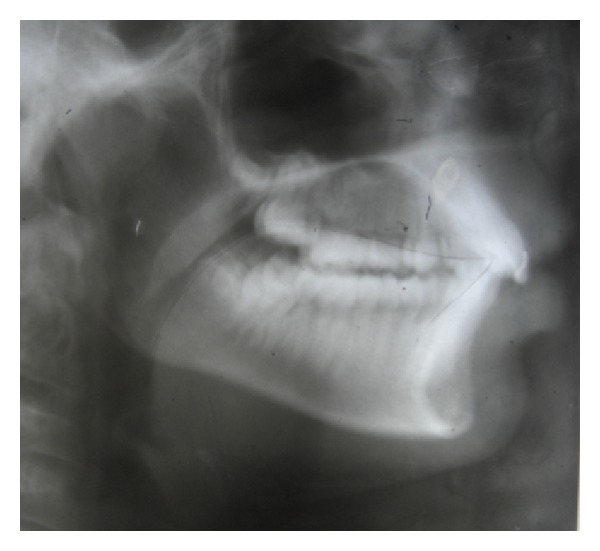
pre-treatment cephalogram showing low positioning of tongue at rest position.

A 13-year-old female patient presented to the Department of Orthodontics and Dentofacial Orthopaedics, Sri Hasanamba Dental College, Hassan, India, with a complaint of forwardly placed upper and lower front teeth. The extraoral examination of the patient showed good facial symmetry, convex profile, acute nasolabial angle, incompetent lips and shallow mentolabial sulcus. Intraoral examination showed class I molar and canine relation on either sides, rotated second premolars in all quadrants, proclined upper and lower anteriors and increased overjet and overbite with anterior traumatic bite. Functional examination suggested that she had tongue thrusting habit which was confirmed by palatography (Figure 3) (palatography involves recording the contact surface of the tongue with the palate and teeth while the patient produces speech sounds such as S or while swallowing). A thin uniform layer of contrasting, precise impression material was applied to the patient's tongue with a spatula. Once the tongue movement (swallowing) was carried out, the palatogram was documented photographically using a surface mirror. The abnormal position of the tongue was also confirmed by tracing the pretreatment lateral cephalogram (Figure 4). The case was diagnosed as bimaxillary dentoalveolar protrusion with simple tongue thrusting habit. It was decided to eliminate the etiology of the malocclusion at the first phase and then proceed to the fixed appliance phase.

Elastomeric separators were placed in the mesial and distal proximal contact areas of maxillary first molars and alginate impressions were taken for upper and lower arches. An HHCA was fabricated in the lab and was cemented to the maxillary first molars of the patient. The patient was asked to constantly pull the bead towards the posterior region of the mouth. She was also advised to make sure that her tongue wedges between the bead and the roof of the mouth when she swallows. She was made aware of the difficulty in pronunciation she may face for a week or so.

**Figure 5:**
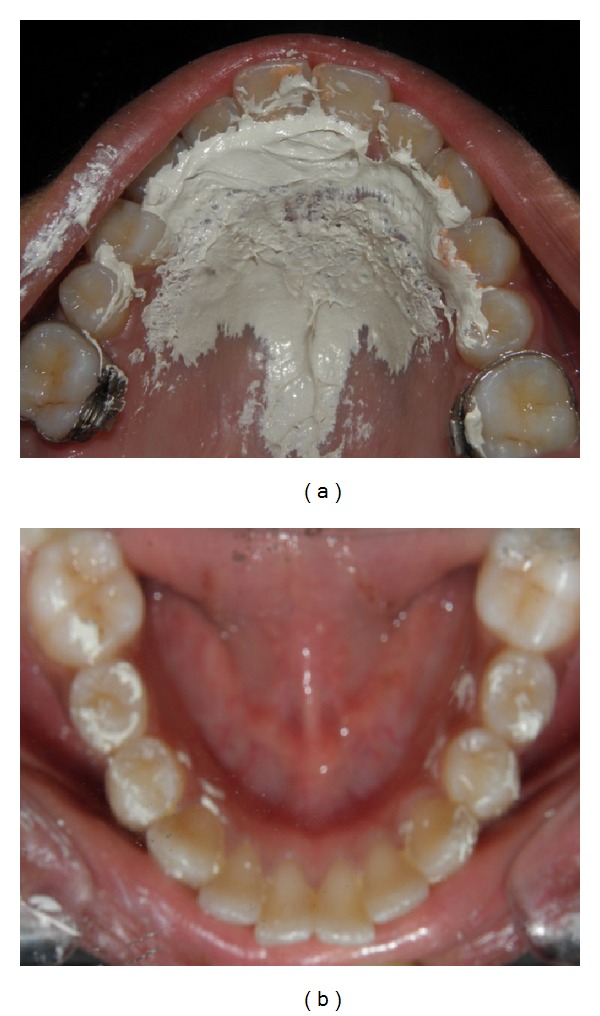
Palatographic analysis six months after the placement of HHCA showing impression material smeared over the maxillary rugae area missing the crown of the incisors indicating that the patient has attained a somatic swallow.

**Figure 6:**
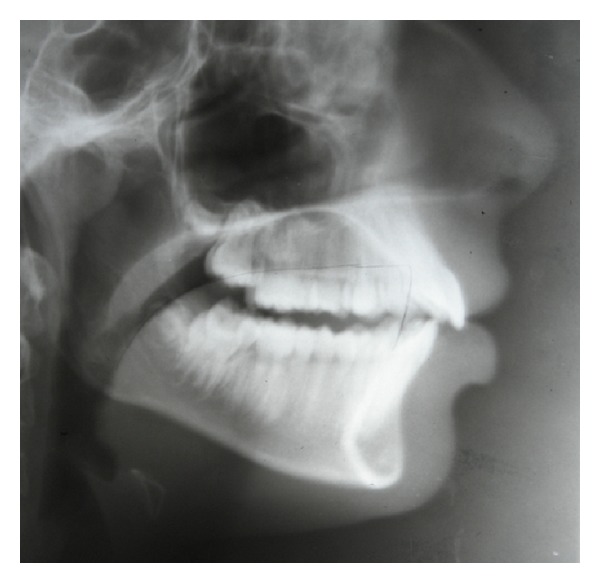
Cephalogram after 6 months of HHCA therapy showing low positioning of tongue at rest position.

The patient was recalled for checkup every month and after 6 months the appliance was removed and checked for the tongue position and swallowing pattern. Functional examination showed that the patient had changed her swallowing from an infantile to mature pattern, which was confirmed by palatography (Figure 5). A lateral cephalogram was taken to confirm the tongue position at rest which showed a more superior and normal tongue posture. The dorsal part of the tongue was resting on the palate and the tip was place behind the upper incisor in the area of the incisive papilla (Figure 6). The HHCA was cemented back to retain the achieved correction and the second phase of correction using fixed appliance was started. This hybrid appliance proved to be highly effective in correcting the tongue thrusting habit in a short span of six months.

## 4. Discussion 

Tongue thrusting is defined as a human behavioral pattern in which the tongue protrudes through the anterior teeth during swallowing, speech and at rest [17]. 

Thumb (digit) sucking usually involves placing the thumb into the mouth and rhythmically repeating sucking contact for a prolonged duration and is considered to be soothing and therapeutic for the person [7]. 

Both of these habits are considered to be normal up to four to five years of age [18]. But it can lead to deleterious effects in the oral cavity if these habits persists beyond the eruption of the permanent teeth. 

Tongue thrust can be *primary*, the etiological factors of which include learned behavior, hyperplastic tonsils, prolonged thumb sucking, nasal congestion and macroglossia, or can be *secondary *to early extraction of deciduous teeth or an anterior open bite [19]. According to Proffit, the anterior tongue position at rest may have greater impact on the tooth position rather than the tongue pressure during thrusting. Hence, the aim of the treatment primarily is to train the tongue to rest in its normal superior position [17]. Elimination of the etiology is the primary and the most important step in the correction of the tongue thrusting habit. Once the cause is determined and eliminated, the tongue thrusting habit is usually dealt in two ways: (1) Muscle retraining—an exercise technique that reeducates the muscles associated with swallowing; (2) mechanical restraining method, where an appliance is placed in the mouth which will prevent the tongue from thrusting forward and thus retrains the tongue to a normal position. Tongue cribs or rakes are valuable mechanical restrainers. Tongue beads placed in the rugae region are conventionally used to retrain the tongue [4–7, 20, 21].

The habit of sucking the finger (or thumb) is considered to be performed for oral gratification and psychological reassurance. Severe thumb sucking can lead to proclination of maxillary anteriors, constriction of the maxilla, retroclination of the mandibular incisors, increased overjet and anterior open bite [22]. Usually, in cases with anterior open bite due to thumb sucking, a secondary tongue thrust develops leading to the exaggeration of the condition. The line of treatment for the prolonged digit sucking involves positive reinforcements, developing a desire in the patient to quit the habit, reminders and appliances which act as a mechanical barrier as well as physical reminders. Appliances consisting of cribs in the anterior region are found to be very effective as reminders as well as physical restrainers [23–26].

The *HHCA* is a hybrid appliance with multipronged advantages. This single appliance can be used to treat both tongue thrusting as well as digit sucking. The bead acts as a training device in tongue thrusting cases, which prevents the low positioning of the tongue and helps to position the tongue in the region of the incisive papilla. In cases with thumb sucking, the bead can serve as a *reminder*. The patient turns the bead which replaces the desire to suck the thumb. 

Another advantage of HHCA is the presence of U-loop in the premolar region which permits the anterior part of the appliance to be repositioned posteriorly. This simple loop allows the appliance to be used during the retraction phase in fixed orthodontic therapy. Hence, the correction of the malocclusion as well as the habit can be done simultaneously. This saves treatment time, as the elimination of habit, which can be the etiology, is advocated before the correction of the malocclusion.

The HHCA can also be used for the correction of posterior crossbites. The posterior leg of the appliance can be expanded and placed into the lingual sheath or soldered. This is particularly useful in patients with constricted maxilla and posterior cross bite due to digit sucking habit. 

The appliance should be retained for another six months after the correction of the habit. 

## 5. Conclusion

HHCA can be effectively used to correct tongue thrusting as well as digit sucking habits. It can act as a device for retraining the associated musculature, a mechanical restrainer and a reminder to discontinue the habit. This appliance gives the flexibility to be used along with the fixed appliance which increases its efficiency as well as reduces the appliance wear time. It can also be used to correct posterior cross bites.
